# One-Year Review in Cardiac Arrest: The 2022 Randomized Controlled Trials

**DOI:** 10.3390/jcm12062235

**Published:** 2023-03-14

**Authors:** Alessio Penna, Aurora Magliocca, Giulia Merigo, Giuseppe Stirparo, Ivan Silvestri, Francesca Fumagalli, Giuseppe Ristagno

**Affiliations:** 1 Department of Pathophysiology and Transplantation, University of Milan, Via Festa del Perdono 1, 20122 Milan, Italy; 2 Department of Anesthesiology, Intensive Care and Emergency, Fondazione IRCCS Ca’ Granda Ospedale Maggiore Policlinico, Via Francesco Sforza 35, 20122 Milan, Italy; 3 Mario Negri Institute for Pharmacological Researches IRCCS, Via Mario Negri 2, 20156 Milan, Italy; 4 Agenzia Regionale Emergenza Urgenza (AREU), Via Campanini 6, 20124 Milan, Italy

**Keywords:** cardiac arrest, randomized controlled trial, cardiopulmonary resuscitation, outcome

## Abstract

Cardiac arrest, one of the leading causes of death, accounts for numerous clinical studies published each year. This review summarizes the findings of all the randomized controlled clinical trials (RCT) on cardiac arrest published in the year 2022. The RCTs are presented according to the following categories: out-of- and in-hospital cardiac arrest (OHCA, IHCA) and post-cardiac arrest care. Interestingly, more than 80% of the RCTs encompassed advanced life support and post-cardiac arrest care, while no studies focused on the treatment of IHCA, except for one that, however, explored the temperature control after resuscitation in this population. Surprisingly, 9 out of 11 RCTs led to neutral results demonstrating equivalency between the newly tested interventions compared to current practice. One trial was negative, showing that oxygen titration in the immediate pre-hospital post-resuscitation period decreased survival compared to a more liberal approach. One RCT was positive and introduced new defibrillation strategies for refractory cardiac arrest. Overall, data from the 2022 RCTs discussed here provide a solid basis to generate new hypotheses to be tested in future clinical studies.

## 1. Introduction

Sudden cardiac arrest is the third leading cause of death in Europe [1]. Mortality, especially after out-of-hospital cardiac arrest (OHCA), remains high despite advances in knowledge and treatments. For this reason, many efforts have been made over the years to improve different aspects of resuscitation, from early recognition of cardiac arrest and activation of an emergency medical system (EMS) to bystander-initiated cardiopulmonary resuscitation (CPR) with early defibrillation, up to the most recent advanced extracorporeal supports in the instance of refractory cardiac arrest [2,3,4].

Appreciable efforts have been made to understand the background and causes of cardiac arrest, as well as the differences in the incidence and outcome among countries, that are related to the variability in data collecting, case-mix, community engagement, EMS organization, quality of treatment, and post-resuscitation care [5]. A 2022 multicenter observational study, the “Swecrit Biobank blood samples from critically ill patients and healthy controls” (SWECRIT), highlighted the differences in patient characteristics and final outcomes between 245 in-hospital (IHCA) vs. 544 OHCA patients [6]. Compared to OHCA, IHCA was more commonly witnessed (85.3% vs. 76.2%) and presented less frequently a cardiac cause (38.8% vs. 69.0%) with a shockable initial rhythm (20.8% vs. 48.2%). All resuscitation timings, known to be associated with long-term outcome, i.e., no-flow, low-flow, time to advanced life support (ALS), and time to return of spontaneous circulation (ROSC), were also confirmed to be dramatically shorter in the instance of IHCA than OHCA (0 vs. 1, 10 vs. 20, 0 vs. 8, 10 vs. 25 min, respectively). Indeed, OHCA presented a significantly higher 30-day mortality (62.2% vs. 49.8%) and a more common poor neurological outcome when compared to IHCA (31.6% vs. 40.0%).

**Figure 1:**
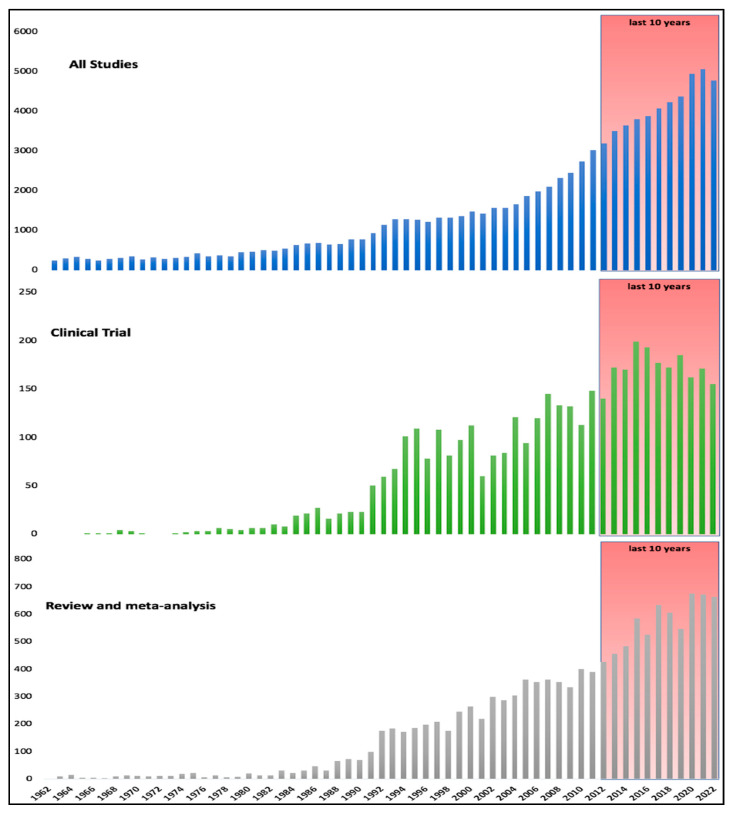
Studies on cardiac arrest published in the last 60 years, with a focus on the last ten years.

Awareness and scientific attention to cardiac arrest has been increased in the last decade, as also represented by the large number of clinical studies in this field. Searching for “cardiac arrest” in Medline, led to 1611 clinical studies and 5524 reviews (both narrative and systematic) and meta-analyses over the last 10 years (Figure 1). Including experimental and basic research, 5415 articles have been published per year over the last 5 years. This review summarizes the findings of all the randomized controlled clinical trials (RCT) on cardiac arrest published in the year 2022. 

## 2. Materials and Methods

The search was conducted in Pubmed and EMBASE in December 2022, with the last update on 9 January 2023. The terms “cardiac arrest OR cardiopulmonary resuscitation” were used as Mesh in the search string, limited to: year 2022 (1 January 2022–31 December 2022); randomized controlled trial; adult age; and English language. Pre-prints were excluded.

**Figure 2:**
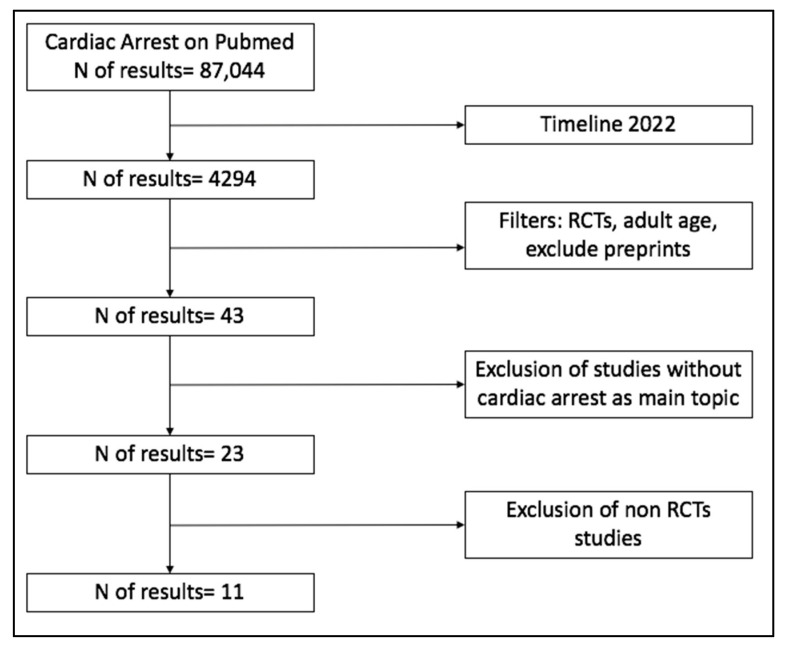
Diagram flow of article selection for inclusion in the review.

Study selection and data/results analysis were performed by two reviewers (A.P., A.M.). Forty-three articles were found, twenty of which were excluded because cardiac arrest was not the main topic of the manuscript. Among the 23 articles focusing on cardiac arrest, 11 were RCTs and are discussed in this review (Figure 2). 

## 3. Results and Discussion of 2022 RCTs

**Figure 3:**
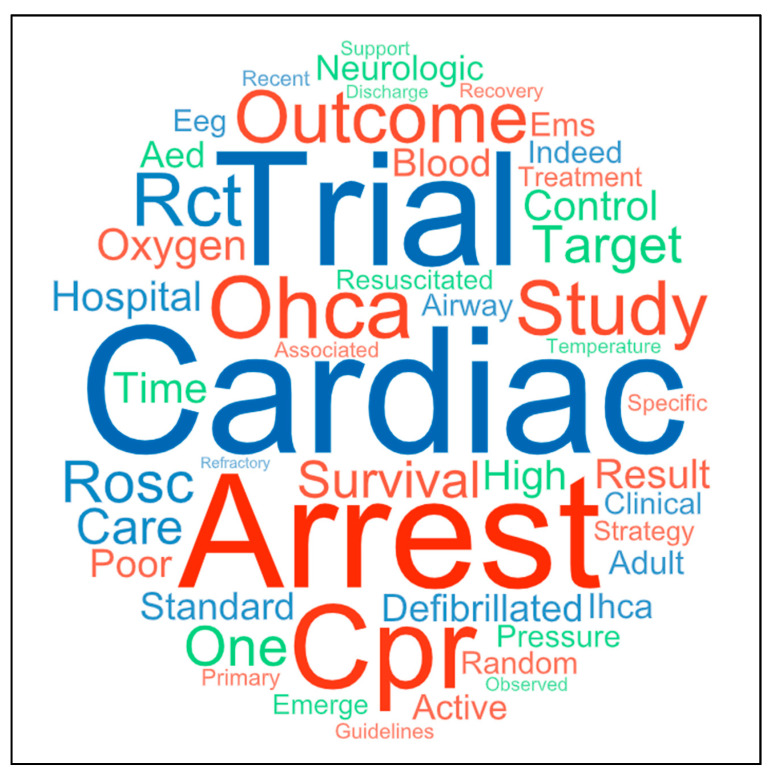
Word cloud of the 50 most common terms present in the 11 randomized clinical trials included in the review.

Figure 3 is the word cloud of the most common terms present in the 11 RCTs included in this review. 

The RCTs are divided according to the following categories: OHCA, IHCA, and post-cardiac arrest care. The main characteristics of the 2022 RCTs in cardiac arrest are reported in Table 1.

### 3.1. Out-of-Hospital Cardiac Arrest

The RCTs discussed are organized according to the chain of survival, represented by the following 6 time-sensitive interventions (i.e., links) to maximize the chance of survival: activation of the emergency response; high-quality CPR; early defibrillation; advanced resuscitation; post-cardiac arrest care; and recovery [7,8].

**Figure 4:**
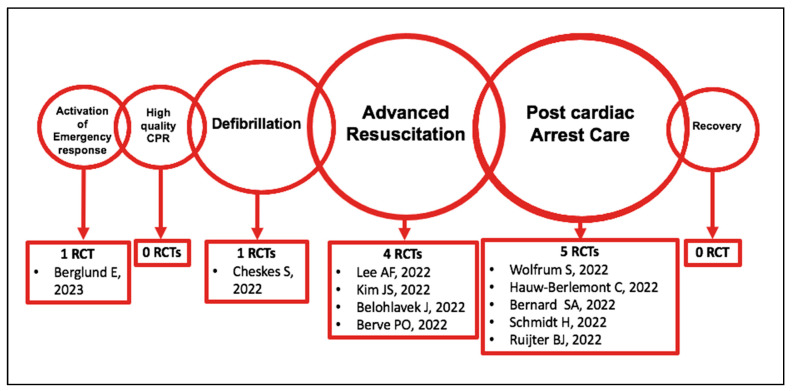
Number of randomized clinical trials published in 2022 for each link of the chain of survival [4,9,15,17,22,30,38,45,51,52,60,63,65].

Figure 4 reports the number of RCTs published in 2022 for each link of the chain of survival.

#### 3.1.1. Activation of the Emergency Response

The recent EuRECA-two epidemiological study on OHCA has reported an average bystander CPR rate of 58%, ranging from a minimum of 13% to a maximum of 82% among countries in Europe [5]. Thus, the European Resuscitation Council (ERC) guidelines have introduced the new concept of “Systems Saving Lives” to emphasize the interconnection between community and EMS as a determinant to improve survival of OHCA [2]. In particular, the community is highly encouraged to implement technologies, e.g., a smartphone app or text message, in order to improve the rate of bystander-initiated CPR and the use of automated external defibrillators (AED) [2]. In response to this encouragement, the “Swedish AED and Mobile Bystander Activation” (SAMBA) trial [9] investigated new strategies to improve early activation of emergency response for cardiac arrest. More specifically, the study evaluated whether a smartphone application for the dispatch of volunteer responders could increase the bystander use of AEDs in patients with OHCA before arrival of EMS or on-duty first responders (fire and police services). When an emergency call for a suspected adult OHCA of non-traumatic cause was received during the daytime, volunteer responders’ early CPR and defibrillation were requested via a smartphone application. In the intervention arm, after acceptance of the app alert, 4 of the 5 activated responders received instructions to collect the nearest available AED while the other one (closest to the cardiac arrest victim) was dispatched to initiate CPR. In the control group, all alerted volunteer responders were instead instructed to go directly to the patient to perform CPR, with no provision of any indication for nearby AEDs. In the 947 randomized OHCAs, AED was similarly attached by volunteer responders in the 13.2% of patients in the intervention arm vs. 9.5% in the control one. Nevertheless, the majority of AEDs was attached by lay volunteers who were not alerted through the smartphone application. No differences were reported in the proportion of bystander-initiated CPR and defibrillation before EMS arrival between the 2 groups (69% vs. 71.6% and 3.7% vs. 3.9% in the intervention and control group, respectively). Before this trial, only analyses on pre–post implementation of community apps for first-responder dispatch were available and anticipated reductions in the response time with volunteers arriving at the OHCA scene prior to EMS in the majority of instances [10]. Surprisingly, the SAMBA trial [9], the first RCT testing the real impact of smartphone alerting technologies to dispatch volunteer rescuers, failed to show any significant increase in volunteer AED use. However, it cannot be excluded that the neutral results of this study might have been confounded by the poor compliance to instructions and contamination of study groups by crossover [9]. Indeed, many volunteer rescuers retrieved and attached an AED even without receiving specific instructions. It is likely that this study was not able to show any benefit of such a technology in a community that already had a high rate of first responders, while it may provide benefit in an area with lower bystander CPR and defibrillation. Thus, designing further RCTs to better evaluate the role of new technologies to engage a community to react promptly in the instance of OHCA needs to be encouraged, especially in areas with known baseline low bystander response, since immediate initiation of CPR has been shown to triple survival in addition to public-access to AEDs [10,11,12].

#### 3.1.2. Defibrillation

Survival after OHCA depends not only on prompt initiation and delivery of high-quality CPR, but also on delivery of effective defibrillation attempts, when required [13].

Conventional defibrillatory pad placement is the antero–lateral (or sternal–apical) one. However, recent CPR guidelines have suggested that in the instance of refractory ventricular fibrillation (VF), a change in the pad position could have been considered [14]. Indeed, this assumption was not supported by any clear scientific evidence, till 2022 when the “Double Sequential External Defibrillation for Refractory Ventricular Fibrillation” (DOSE VF) trial [15] was published. In this trial, refractory VF, a common condition observed in approximately 20% of patients [16], was defined as VF or pulseless ventricular tachycardia that persisted after three standard defibrillation attempts. In this instance, a double sequential external defibrillation (DSED, i.e., two shocks delivered sequentially within 1 sec interval) and a vector-change (VC) defibrillation (i.e., switching of the defibrillation pads from the anterior–lateral to the anterior–posterior position) have been investigated as new interventions to improve outcome in this specific population. Thus, among the 405 randomized patients: 136 continued to receive standard defibrillation attempts through the original pads position; 125 were defibrillated with the VC approach; and 144 received DSED after paramedics applied a second set of defibrillation pads connected to a second defibrillator. The primary outcome, i.e., survival to hospital discharge, was significantly higher in the DSED and VC groups compared to the standard approach. Indeed, 30.4% of patients in the DSED and 21.7% in the VC survived, compared to 13.3% in the standard group. In addition, termination of VF was also significantly higher after the application of either one of the two new defibrillation strategies compared to the standard one, i.e., 84% after DSED, 79.9% after VC, and 67.6% after standard defibrillation. ROSC was also greater with the two modified defibrillation approaches compared to the standard one. Finally, DSED but not VC defibrillation was also associated with a significantly higher percentage of patients achieving a good neurologic recovery in comparison with standard defibrillation. Indeed, a modified Rankin score < 3 was present in 27.4% of patients who received DSED, compared to 16.2% treated with VC and 11.2% with standard defibrillation. This trial had the limitation of having been stopped early because of the COVID-19–related challenges in EMS rescue interventions, not achieving the planned sample size. In addition, data from an initial pilot trial were included in the final analyses, and no data on post-resuscitation care or follow-up were collected. These limitations notwithstanding, this trial is of great importance because it was one of the few positive trials in cardiac arrest over the past decade, offering new insights for clinical practice as well as suggestions for future studies [15]. Indeed, the results from this trial suggest that changing the defibrillation pad placement may increase the likelihood of terminating VF by increasing the amount of myocardial mass reached by the electrical current during the shock. Increasing the overall defibrillation energy may also play a role in increasing the success of defibrillation, as anticipated by the DSED approach. This last hypothetical explanation re-opens the past controversial issue on the relationship between higher energy shocks and worse post-resuscitation myocardial dysfunction and outcome. Thus, while the VC approach is feasible and easy to implement in the current practice, the DSED intervention appears far from routine use. Future studies are needed, as well as practical procedures to overcome limitations, such as adequate personnel training, availability of two defibrillators, and capability to deliver two electrical shocks with a consistent, but still unknown, intervening pause.

#### 3.1.3. Advanced Life Support

This is one of the CPR areas in which research and consequent scientific publications have concentrated the greatest efforts in 2022, with a special focus on airway management, pharmacological interventions, technological developments, and implementation of extracorporeal supports.

##### Airway Management

Pre-hospital advanced airway management with either endotracheal intubation (ETI) or supraglottic airway (SGA) insertion in patients with OHCA remains controversial. The most recent guidelines prompted for either bag-mask ventilation or an advanced airway strategy during CPR, based on the rescuers’ skill. Indeed, when an advanced airway is used, SGA is suggested in settings with a low ETI success rate, leaving the use of endotracheal tubes to professional rescuers with high ETI success rates, i.e., >95% within two attempts [14]. The “Supraglottic Airway Device vs. Endotracheal Intubation” (SAVE) trial [17] compared the effectiveness of ETI vs. SGA in adult patients with non-traumatic OHCA, targeting sustained ROSC as the primary outcome. EMS systems were cluster-randomized to ETI or SGA, and a total of 936 patients (517 in the ETI group and 419 in the SGA group) were included in the primary analysis. The first-attempt airway success rate was similar in the two groups, i.e., 77% for ETI and 83% for SGA. Sustained ROSC was achieved in 26.9% of patients after ETI vs. 25.8% after SGA. No differences in survival to hospital discharge and neurological recovery were observed [17]. Thus, the SAVE trial, by confirming the equivalency of these two airway strategies in terms of ROSC and long-term outcome, consistently with the earlier AIRWAYS 2 trial [18], supports current clinical recommendations [14]. However, it has to be mentioned that the study protocol allowed for only a single ETI attempt, and therefore we do not know if the EMS teams’ skill in performing this procedure was in line with current recommendations (e.g., ETI success > 95%) or was lower, ultimately reducing any eventual benefit from ETI during CPR. Indeed, in this study, ETI was confirmed to be associated with prolonged scene time intervals and time for airway insertion [18], known to be a likely determinant of worse outcome [19]. Interestingly, the probability of pre-hospital ROSC was, however, statistically higher in the ETI group than in the SGA group, especially in patients with non-shockable cardiac arrest. Thus, the SAVE trial anticipates that cardiac arrests of non-cardiac origin may benefit from pre-hospital management with ETI, although this issue would require further studies.

##### Pharmacological Interventions

For more than 60 years, CPR guidelines have continued to recommend adrenaline as the main drug for CPR [14], supported also by the results of the earlier PARAMEDIC 2 study, which has finally demonstrated a significantly higher long-term survival after adrenaline compared to placebo in more than 8000 OHCA patients [20].

Vasopressin has been an alternative vasopressor to adrenaline in the past, mainly appreciated for the lack of catecholamine effects. However, past trials have shown no benefits of vasopressin for ROSC or neurologic outcome over the standard dose of adrenaline, such that its use in CPR was abandoned [21]. The “Augmented-Medication CardioPulmonary Resuscitation” (AMCPR) trial [22] is a new pilot RCT on the use of vasopressin during CPR published in 2022. A total of 148 adult non-traumatic OHCA patients with shockable presenting rhythm, who presented a diastolic blood pressure (DBP) < 20 mmHg after insertion of an arterial line at admission to the emergency department, were randomized to receive 40 UI vasopressin or placebo in addition to adrenaline. The primary outcome of sustained ROSC was not different between the two groups, i.e., 36.5% in vasopressin vs. 32.4% in the placebo group. Survival to hospital discharge and good neurologic recovery were also not different between groups. Nevertheless, median DBP during resuscitation was significantly higher in patients receiving vasopressin than in those treated with placebo, likely increasing coronary perfusion pressure during CPR [22]. Thus, this study does not support the need to modify current recommendations by reintroducing vasopressin as an alternative drug for CPR. However, there are other studies that have shown benefits from a combination of vasopressin with corticosteroids for IHCA on a short-term outcome, but with more uncertainty regarding survival and neurological recovery [23,24,25].

##### Extracorporeal CPR (ECPR)

ECPR provides artificial circulation, oxygenation, and carbon dioxide removal through an extracorporeal pump and serves as a bridge to the definitive treatment of reversible OHCA causes [26]. Refractory OHCA, defined as the failure to achieve ROSC despite conventional ALS, is associated with poor prognosis [27]. With increasing frequency of use and evidence of improved outcomes with ECPR for refractory OHCA, many centers are implementing this approach, and new studies are taking place in this area. A great stimulus was given by the results of the recent ARREST trial [3] that demonstrated how this support significantly improved survival to hospital discharge for selected patients compared with standard ALS in the presence of a well-implemented EMS system, allowing for ECPR initiation within 1 h from collapse.

In 2022, the Prague trial [4] evaluated the impact of an early invasive approach in 256 refractory OHCAs, including early intra-arrest transport with ongoing mechanical compression to the closest cardiac arrest center for rapid institution of ECPR and an immediate invasive assessment and treatment, on survival with good neurological outcome in comparison to standard ALS. Survival with a favorable neurologic outcome at 180 days was similar between the two strategies, occurring in 31.5% of patients undergoing the invasive strategy vs. 22% in whom standard ALS was continued. The trial was stopped prematurely when prespecified criteria for futility were met and thus was underpowered to detect a statistically significant difference for the primary outcome. In addition, the single-center design with limited enrollment and risk of crossover represents another important limitation of this trial. Nevertheless, a significantly improved 30-day neurologic recovery was observed in favor of the invasive strategy with an odds ratio of 1.99 (1.11–3.57) [4].

In a subsequent post-hoc analysis of the Prague trial, authors reported that in the presence of an initial shockable rhythm, treatment with the invasive approach was associated with a neurologically favorable survival for 180 days [28]. Indeed, survival with good neurological recovery between shockable and non-shockable initial rhythms was significantly different, i.e., 49% vs. 8% in the invasive strategy and 33% vs. 2% in the standard ALS. Another secondary analysis also reported that ECPR improved 180-day survival in patients who did not achieve pre-hospital ROSC [29]. In fact, the overall 180-day survival in patients without pre-hospital ROSC increased from 1.2% in those receiving conventional ALS to 23.9% in those treated with ECPR [29]. Rapid deployment of veno-arterial extracorporeal circulation during ongoing CPR is, therefore, a promising approach for patients with refractory non-traumatic OHCA and has demonstrated improved outcomes in selected patients with favorable cardiac arrest circumstances, although limited data exist regarding the best practices for ECPR [14]. Thus, a recent 2022 international expert consensus, attempting to detail best practices for ECPR initiation following OHCA, has been released in 2022 [26].

##### Adjunct Treatments during CPR

The “cardiopulmonary resuscitation with active compression–decompression” (ACD-CPR) trial [30] was an RCT testing of whether an active mechanical decompression up to 30 mm above the patient’s sternal resting position would generate better hemodynamics than standard mechanical CPR, assessed in terms of maximum end-tidal CO_2_ during compression (EtCO_2_). A total of 210 OHCA patients intubated and with monitored capnography were randomized to ACD or standard CPR. No differences in EtCO_2_ (29 vs. 29 mmHg), nor in blood pressure or oxygen saturation of cerebral tissue (SctO_2_), were observed between the two groups during CPR. However, the authors highlighted that in 48 patients who received a complete active decompression, a significantly higher SctO_2_ than control patients, i.e., 58% vs. 55%, was present in more than half of the observations [30]. Although these post-hoc results may appear of interest because they are in support of the use of mechanical ACD, when properly delivered, they should be interpreted with caution since they are derived from partial data obtained only from a relatively small portion of the study population. In addition, statistical differences do not always parallel clinical significance, as in this instance, where a 3% absolute difference in SctO_2_ was described.

Active decompression (AD) improves lower intrathoracic pressures in the decompression phase, favoring a venous return to the heart and subsequent cardiac output [31]. Clinical studies on manual ACD-CPR have shown equal or improved EtCO_2_ and survival with this approach compared to standard CPR [32,33]. Nevertheless, the increased fatigue to provide AD was an important restraint [34], while a mechanical ACD-CPR device could have overcome this issue. The ACD-CPR trial, however, failed to show any additional benefit by mechanical AD compression compared to standard mechanical CPR. The most important limitation of this study, besides the modest sample size, was that complete AD was not reached consistently due to the detachment of the suction cup from the chest, preventing the intervention device from fully lifting the chest to deliver a complete AD, this might have been the reason for the neutral result [30]. Thus, future studies with optimized devices are necessary to investigate the clinical impact of mechanical ACD-CPR.

### 3.2. In-Hospital Cardiac Arrest

Despite IHCA representing a not-rare event characterized by high mortality, the number of RCTs focusing on this topic is few [1]. Furthermore, the year 2022 was characterized by a limited scientific interest in IHCA, with only one RCT that investigated the still-debated temperature control management (TTM) in this population. After almost a decade of hypothermia practice, in 2013, the TTM trial showed no difference in all-cause mortality or 6-month neurological function between patients who received temperature control to a target of 33 °C versus a target of 36 °C in 939 OHCA patients [35]. More recently, the TTM-2 trial confirmed the above results in 1850 comatose OHCA survivors who were randomized to temperature controlled at 33 °C vs. fever prevention, defined as body temperature > 37.7 °C [36]. Based on these results and on a recent ILCOR metanalysis, ERC and the European Society of Intensive Care Medicine (ESICM) guidelines recommended continuous monitoring of core temperature with only active prevention of fever (temperature > 37.7 °C) for at least 72 h in comatose patients after cardiac arrest [37].

In 2022, the “Hypothermia After Cardiac Arrest in-hospital” (HACAinhospital) trial [38] was conducted to determine the effect of hypothermic temperature control (32–34 °C) for 24 h vs. normothermia (37.0 °C ± 0.9 °C) on mortality and neurological outcome in 249 patients resuscitated from IHCA. No differences in any outcome were reported. Indeed, 180-day mortality was 72.5% vs. 71.2% in the hypothermia vs. normothermia strategy, while patients with a Cerebral Performance Category of 1 or 2 were 22.5% vs. 23.7%, respectively [38]. Thus, these results confirm and support the absence of a need to apply hypothermic temperature control in comatose patients resuscitated from IHCA. In this view, the HACAinhospital trial may appear as another publication further reinforcing the evidence of the lack of effects of hypothermic control in ameliorating the outcome of comatose post-cardiac arrest patients. The key question is, therefore, whether we still need other studies on hypothermic TTM or not. Overall, there is moderately strong evidence of no additional benefit of this intervention in comparison to normothermic TTM in OHCA, while studies on IHCA are only a few numbers, of limited size, and with conflicting results [39]. Thus, large and powered RCTs are likely needed to make a solid recommendation for this specific population of patients after IHCA. Nevertheless, it is probably time to investigate hypothermic TTM not alone but in conjunction with more comprehensive interventions potentially impacting outcomes, such as sedation level, duration, and hemodynamics (i.e., with more sharp pressure targets compared to those discussed in the following paragraphs) and in specific populations in which cooling might still have a beneficial role, as suggested by recent reports, i.e., patients resuscitated after long CPR with high epinephrine administration or with severe post-cardiac arrest illness [40,41].

### 3.3. Post-Cardiac Arrest Care

The treatment of cardiac arrest is not limited to obtaining ROSC, but also encompasses the prompt initiation of standardized post-cardiac arrest care to ensure long-term survival with adequate neurological recovery [25,42]. The main updates in the latest ERC-ESICM post-cardiac arrest guidelines include: the need for early coronary angiography; blood pressure targets; treatment of seizures; temperature management; general intensive care management; and prognostication. Most of these points have seen new RCTs released in 2022.

#### 3.3.1. Early vs. Delayed Percutaneous Coronary Intervention

The most common cause of sudden cardiac death is ischemic cardiovascular disease, mostly acute coronary artery occlusion. Guidelines recommend performing an emergent coronary artery angiography (CAG) in survivors of sudden cardiac death with ST-segment elevation at the post-ROSC ECG. In patients with cardiac arrest without ST-segment elevation, the benefit of an emergency CAG is still a matter of debate since the rate of acute coronary artery lesion is much lower, i.e., <20% [43]. Thus, in these patients, emergent cardiac catheterization laboratory evaluation should be considered if there is an estimated high probability of acute coronary occlusion, i.e., hemodynamic and/or electrical instability [25,44]. The “Emergency vs. Delayed Coronary Angiogram In Survivors of Out-of-Hospital Cardiac Arrest” (EMERGE) trial [45] randomized 279 adult survivors from OHCA without ST-segment elevation on ECG to either emergency vs. delayed CAG, i.e., 48 to 96 h after ROSC. The primary outcome was a 180-day survival rate with good neurological recovery. The mean time delay between randomization and CAG was 0.6 h in the emergency CAG group and 55.1 h in the delayed approach. The 180-day survival rate with good neurological outcome was 34.1% in the emergency CAG group vs. 30.7% in the delayed one. There was also no difference in all secondary outcomes, which included occurrence of shock or malignant arrhythmia within 48 h, level of myocardial dysfunction, overall survival, neurological recovery at intensive care unit (ICU) discharge, and hospital length of stay [45]. This 2022 RCT confirmed that a strategy of emergency CAG was not proven to be better than a strategy with a delayed CAG, consistent with results from earlier published studies and a recent systematic review and meta-analysis [45,46,47,48,49]. However, although the data from the EMERGE study may appear reasonable because it aligned with available evidence, we have to acknowledge that such a neutral result has been likely due to the extreme underpower of the study to adequately assess the endpoints. Indeed, an absolute difference of 10% between the 2 study groups was foreseen with 970 patients, while approximately only one-quarter of this planned number was enrolled. Since with such a small sample size, a 4.4% absolute difference in the primary endpoint was already observed, the readers are allowed to criticize the study results and take-home message, which might have been totally different if the population enrollment target was achieved.

#### 3.3.2. Oxygen Target

Determination of the optimal post-cardiac arrest oxygen target is still a knowledge gap. Animal data and clinical studies have indicated that the administration of 100% oxygen during the early post-arrest period leads to severe hyperoxia, increased neurological injury, and poor clinical outcome [50]. Thus, current guidelines recommend titrating the inspired oxygen to achieve an arterial oxygen saturation of 94–98% or arterial partial pressure of oxygen (PaO_2_) of 75–100 mmHg after ROSC once peripheral oxygen saturation (SpO_2_) can be measured reliably [39]. In 2022, two RCTs investigated new oxygen therapy strategies to establish the optimal PaO_2_ targets in post-ROSC management: the “Blood Pressure and Oxygenation Targets in Postresuscitation Care” (BOX) trial [51] and the “Reduction of Oxygen After Cardiac Arrest” (EXACT) trial [52].

The BOX trial [51] randomized 789 comatose OHCA patients, with a 2-by-2 factorial design, to a restrictive PaO_2_ of 68–75 mm Hg or a liberal PaO_2_ of 98–105 mm Hg. Patients were also assigned to two blood-pressure targets, as reported in the following section of this review. The primary outcome, which was a composite of death from any cause or hospital discharge with a poor neurological function, i.e., CPC of 3–4, within 90 days, occurred likewise in 33.9% of patients treated with a liberal oxygen target vs. 32% with a restrictive approach. In parallel, the median neuron-specific enolase level at 48 h was 17 μg/L in the restrictive target group and 18 μg/L in the liberal one. The incidence of adverse events was also similar in the two groups. Thus, this trial was also a neutral study, resulting in a similar outcome after either higher or lower PaO_2_ was targeted in comatose patients resuscitated after cardiac arrest [51]. The potential pathophysiological link between brain injury and oxygenation seems to occur in the early period after ROSC and to be driven by reperfusion-led mitochondrial dysfunction and tissue inflammation [50,53]. However, in the BOX trial, a substantial separation of PaO_2_ values started later after ROSC, i.e., approximately 2 h after ICU admission. This time window was probably too long to avoid reperfusion injury to start.

This doubt might have been resolved by the concurrent EXACT trial [52], specifically conducted to determine whether reducing the fraction of inspired oxygen (FiO_2_) immediately following resuscitation would improve survival to hospital discharge. Four hundred and twenty-eight adult OHCA patients of presumed cardiac cause, with an established advanced airway and a SpO_2_ of at least 95% while receiving more than 10 L/min of oxygen or FiO_2_ of 100%, were randomized to oxygen titration to achieve a target of SpO_2_ of 90–94% in the intervention group or 98–100% in the control one, until ICU arrival. Practically, in the intervention group, oxygen was initially reduced to 4 L/min via an oxygen reservoir bag or an oxygen/air mix setting if the patient was mechanically ventilated and then further titrated to maintain an SpO_2_ of 90–94%. Patients randomized to receive standard care were treated with high-flow oxygen, i.e., >10 L/min of oxygen via an oxygen reservoir bag or FiO_2_ of 100% if mechanically ventilated, and then underwent oxygen titration to maintain an SpO_2_ of 98–100%. Unfortunately, this trial was stopped early due to the COVID-19 pandemic, leading to only approximately one-third of the pre-planned sample size. Overall, 38.3% of patients ventilated with an SpO_2_ target of 90–94% survived to hospital discharge compared to 47.9% of those with higher SpO_2_ (*p* = 0.05). In addition, the incidence of hypoxic episodes prior to ICU was significantly higher in the lower SpO_2_ target (31.3%) than in the standard one (*p* < 0.001) [52]. Surprisingly, the EXACT trial was a negative study with results contrasting previous findings of an association between hyperoxia during the early post-ROSC phase and increased neurological injury in animal and adult studies [54,55,56]. This study concluded that targeting an oxygen saturation of 90–94%, compared with 98–100%, until ICU admission did not significantly improve survival to hospital discharge, while it increases the risk of harm due to pre-hospital hypoxic events. Nevertheless, the lack of accurate oxygen titration needs to be acknowledged as a potential bias [52].

#### 3.3.3. Hemodynamic Targets

A central part of goal-directed post-resuscitation care is maintaining adequate organ perfusion pressure, although evidence for specific blood-pressure targets is limited. Blood pressure is actively managed as part of most ICU protocols to deliver sufficient perfusion pressure to vital organs, such as the brain, heart, and kidneys. More specifically, mean arterial pressure (MAP) is one of the main determinants of cerebral blood flow [55]. In addition, in many post-cardiac arrest patients, cerebral flow autoregulation is impaired, and thus cerebral flow may be MAP-dependent with an increased risk of cerebral hypoperfusion or hyperemia [57]. Earlier RCTs, comparing low and high blood pressure targets, i.e., 65–75 mmHg to 80–100 mmHg [58,59], whilst documenting safety of higher MAP targets, failed to report any clear improvements in surrogate markers of brain injury. Several other observational studies found, instead, that hypotension was associated with poor outcome [42]. The BOX trial [60], besides the oxygenation targets, randomized the population to two blood pressure levels, i.e., a low MAP of 63 mmHg and a high MAP of 77 mmHg. A primary-outcome event of death of poor neurological outcome occurred in 34% of patients in the high-target group vs. 32% in the low-target one. Thus, this study also showed that targeting a low or high MAP in patients resuscitated from cardiac arrest did not result in significantly different outcomes. Nevertheless, the mean difference in MAP between the groups was only 10.7 mmHg and therefore was lower than expected (i.e., 14 mm Hg); this limits extrapolation of the findings to higher or lower MAP targets than those used in this trial. In addition, the overall neurological recovery was excellent, with a median CPC of 1, making this population likely too good to be able to show differences in outcome [60]. Thus, future studies on hemodynamic targets are still required, and especially studies targeting sharper MAP classes, with a clear separation among the selected levels of pressure, thus providing a clear answer to the readers and avoiding extrapolations of evidence from study groups largely overlapping each other. The same comment applies to the above-reported studies focusing on post-ROSC oxygen targets.

#### 3.3.4. Prevention of Fever and TTM

Fever is common during the first 2–3 days after cardiac arrest and has been associated with worse outcome in observational studies [61], especially the rebound hyperthermia after TTM [62]. For this, CPR guidelines recommend active fever prevention up to 72 h after ROSC [37]. Nevertheless, clinical data on this recommendation were lacking. Indeed, whether fever contributes to poor neurological outcome or is just a marker of severe brain injury remains unknown. In the attempt to provide a response to this knowledge gap, the BOX trial randomly assigned comatose patients after OHCA of presumed cardiac cause to a device-based temperature control for either 36 or 72 h [63]. More specifically, the temperature was targeted at 36 °C for 24 h followed by a target of 37 °C for either 12 or 48 h (i.e., the total intervention times were 36 or 72 h). A total of 393 patients were randomly assigned to temperature control for 36 h, while 396 patients to temperature control for 72 h. No differences in primary outcomes were observed between the two interventions, i.e., death or severe disability or coma. At 90 days after randomization, a primary endpoint event occurred in 32.3% vs. 33.6% and mortality in 29.5% vs. 30.3% in the 36 h vs. 72 h group, respectively. Thus, this RCT does not yield clear inputs in modifying current clinical practice while further reinforcing the need to redirect research on post-cardiac arrest towards other and possibly more comprehensive interventions to improve outcome, rather than persisting on TTM alone, as in the past twenty years.

#### 3.3.5. Seizure Treatment

The presence of unequivocal seizures on EEG during the first 72 h after ROSC is an indicator of poor prognosis. For this, guidelines recommend using electroencephalography (EEG) to diagnose seizures in patients with clinical convulsions and monitor treatment effects [25]. More specifically, rhythmic and periodic EEG patterns that may reflect seizures have been reported in 10–35% of comatose patients after cardiac arrest and have generally been associated with a poor neurologic outcome [64]. If rhythmic and periodic EEG patterns should be treated with antiseizure medications, with the goal of improving the neurologic outcome, is unclear. The “Treatment of Electroencephalographic Status Epilepticus after Cardiopulmonary Resuscitation” (TELSTAR) trial [65] assessed whether intensive, stepwise antiseizure and sedative treatment to suppress rhythmic and periodic EEG patterns detected in continuous EEG monitoring would reduce the incidence of poor neurologic outcome at 3 months in adult comatose patients after cardiac arrest, in contrast to standard care. One-hundred and seventy-two patients were randomly assigned to a stepwise strategy of antiseizure medications to suppress this activity for at least 48 consecutive hours plus standard care (antiseizure-treatment group) or to standard care alone (control group). The stepwise treatment strategy in the intervention group consisted of: step 1, first antiseizure drug plus a first sedative agent; step 2, second antiepileptic drug plus a second sedative agent; and step 3, a high-dose barbiturate. Rhythmic or periodic EEG activity was detected with a median of 35 h after cardiac arrest. Although a complete suppression of rhythmic or periodic EEG activity for 48 consecutive hours occurred in 56% in the antiseizure-treatment group vs. 2% in the control group, patients with a poor neurological outcome were equivalent in the two management approaches, i.e., 90% vs. 92%, respectively. Mortality was also similar, i.e., 80% vs. 82% in the antiseizure-treatment group vs. control, respectively [65]. The conclusion was that a stepwise antiseizure treatment did not yield any benefit in terms of outcome, despite suppressing the malignant EEG pattern.

**Table 1:** Main characteristics of RCTs published in 2022.

RCT	Population	Intervention	Number of Patients	Primary Outcome	Secondary Outcomes (Main)
SAMBA [9] NCT02992873	OHCA	Intervention: volunteer responders dispatched to the nearest public AED on the way to the OHCA Control: volunteer responders dispatched to perform CPR	947: - 461, intervention - 486, control	Bystander AED attachment: - 13.2%, intervention - 9.5%, control RR (95%CI): 1.40 (0.97–2.01)	Bystander CPR: - 69.0%, intervention - 71.6%, control RR (95%CI): 0.96 (0.89–1.05) Bystander defibrillation: - 3.7%, intervention - 3.9%, control RR (95%CI): 0.94 (0.50–1.79)
DOSE VF [15] NCT04080986	OHCA (Refractory VF)	Standard defibrillation (std) Double Sequential External Defibrillation (DSED) Vector Change defibrillation (VC)	405: - 136, standard - 144, VC - 125, DSED	Survival to hospital discharge: - 13.3%, standard - 21.7%, VC RR: 1.71 (1.01–2.88) vs. std - 30.4%, DSED RR: 2.21 (1.33–3.67) vs. std	Termination of VF: - 67.6%, standard - 79.9%, VC RR 1.18 (1.03–1.36) vs. std - 84%, DSED RR 1.25 (1.09–1.44) vs. std ROSC: - 26.5%, standard - 35.4%, VC RR 1.39 (0.97–1.99) vs. std - 46.4%, DSED RR 1.72 (1.22–2.42) vs. std Good neurological recovery at hospital discharge: - 11.2%, SD - 16.2%, VC RR 1.48 (0.81–2.71) - 27.4%, DSED RR 2.21 (1.26–3.88)
SAVE [17] NCT02967952	OHCA	Endotracheal intubation (ETI) Supraglottic airway (SGA)	936: - 517, ETI - 419, SGA	Sustained ROSC: - 26.9%, ETI - 25.8%, SGA OR 1.02 (0.98–1.06)	Pre-hospital ROSC: - 10.6%, ETI - 6.4%, SGA OR 1.04 (1.02–1.07) Survival to hospital discharge: - 8.5%, ETI - 8.4%, SGA OR 1.00 (0.94–1.06) Good neurological recovery: - 3.9%, ETI - 4.8%, SGA OR 0.99 (0.94–1.03)
AMCPR [22] NCT03191240	OHCA	Intervention: vasopressin + epinephrine Control: epinephrine	148: - 74, intervention - 74, control	Sustained ROSC: - 36.5%, intervention - 32.4% control RR 0.94 (0.74–1.19)	Survival to hospital discharge: - 8.1%, intervention - 8.1%, control RR 1.00 (0.91–1.10) Good neurological recovery: - 0%, intervention - 0%, control RR 1.00 (1.00–1.00)
Prague OHCA [4] NCT01511666	OHCA	Invasive strategy: mechanical compression + intra-arrest transport + ECPR + immediate invasive assessment and treatment. Standard strategy: ACLS	256: - 124, invasive - 132, standard	Survival with good neurological recovery at 180 days: - 31.5%, invasive - 22%, standard OR 1.63 (0.93–2.85)	30-day survival with good neurological recovery: - 30.6%, invasive - 18.2%, standard OR 1.99 (1.11–3.57) 30-day cardiac recovery: - 43.5%, invasive - 34.1% standard OR 1.49 (0.91–2.47)
ACD-CPR [30] NCT02479152	OHCA	Mechanical active compression-decompression (ACD) Conventional mechanical compression	210: - 101, ACD - 109, conventional	Maximal end-tidal CO_2_: - 29 mmHg, ACD - 29 mmHg, conventional	Arterial blood pressure: - 111/21 mmHg, ACD - 101/18 mmHg, conventional Cerebral saturation: - 111/21 mmHg, ACD - 101/18 mmHg, conventional CPR-related injuries: - 54.8%, ACD - 57.3%, conventional ROSC: - 47%, ACD - 50%, conventional Survival: - 8%, ACD - 6%, conventional
HACAinhospital [38] NCT00457431	IHCA	Hypothermic temperature control (32–34 °C) Normothermia	249: - 126, hypothermia - 123, normothermia	180-day mortality: - 73%, hypothermia - 71%, normothermia RR 1.03 (0.79–1.40)	In-hospital death: - 63%, hypothermia - 58%, normothermia RR 1.11 (0.86–1.46) 180-day good neurological recovery: - 22.5%, hypothermia - 23.7%, normothermia RR 1.04 (0.78–1.44)
EMERGE [45] NCT02876458	OHCA	Emergency CAG Delayed CAG (48–96 h)	279 - 141, emergency CAG - 138, delayed CAG	180-day survival with good neurological recovery: - 34.1%, emergency CAG - 30.7%, delayed CAG HR 0.87 (0.65–1.15)	180-day survival: - 36.2%, emergency CAG - 33.3%, delayed CAG HR 0.86 (0.64–1.15) Occurrence of shock during first 48 h - 38.8%, emergency CAG - 39.8, delayed CAG HR 1.03 (0.76–1.39) 90-day good neurological recovery: - 28.4%, emergency CAG - 24.6%, delayed CAG HR 0.86 (0.64–1.14) 180-day LVEF: - 60%, emergency CAG - 57.5%, delayed CAG Hospital length stay: - 7 d, emergency CAG - 5 d, delayed CAG
EXACT [52] NCT03138005	Post-cardiac arrest (OHCA)	Intervention: spO_2_ 90–94% Control: spO_2_ 98%–100%	425: - 214, intervention - 211, standard	Survival to hospital discharge: - 38.3%, intervention - 47.9%, standard OR 0.68 (0.46–1.00)	Hypoxic episode prior to ICU: - 31.3%, intervention - 16.1%, standard OR 2.37 (1.49–3.79) Pre-ICU re-arrest: - 12.7%, intervention - 10%, standard OR 1.30 (0.71–2.38)
BOX [51,60,63] NCT03141099	Post-cardiac arrest (OHCA)	Blood pressure target: - 63 mmHg, low - 77 mmHg, High PaO_2_ target: - 68–75 mmHg, restrictive - 98–105 mmHg, liberal Device-based temperature control: - 36 h - 72 h	789: Blood pressure target: - 396, low - 393, high Oxygen target: - 394, restrictive - 395, liberal Temperature control: - 393, 36 h - 396, 72 h	Death or severe disability or coma: Blood pressure target: - 34%, high - 32%, low HR 1.08 (0.84–1.37) Oxygen target: - 33.9%, liberal - 32%, restrictive HR 0.95 (0.75–1.21) Temperature control: - 32.3%, 36 h - 33.6%, 72 h HR 0.99 (0.77–1.26)	90-day death 90-day neurological recovery 48-h neuron-specific enolase level No significant difference in any secondary outcomes in any target groups
TELSTAR [65] NCT02056236	Post-cardiac arrest (OHCA)	Intervention: stepwise strategy of antiseizure medications Control: standard care	172: - 88, intervention - 84, control	Poor neurological recovery at 3 months: - 90%, intervention - 92%, control Risk difference 2 (−7 to 11)	Death at 3 months: - 80%, treatment - 82%, control Rik difference 3 (−9 to 14) Length of stay in ICU: - 8.7 d, intervention - 7.5 d, control Duration of mechanical ventilation: - 7.8 d, intervention - 6.6 d, control

OHCA: out-of-hospital cardiac arrest; ACLS: advanced cardiac life support; AED: automated external defibrillator; CAG: Coronary angiography; CI: confidence interval; CPR: cardiopulmonary resuscitation; DSED: double sequential external defibrillation (rapid sequential shocks from two defibrillators); ECPR: extracorporeal cardiopulmonary resuscitation; EMS: emergency medical service; ETI: endotracheal intubation; HR: hazard ratio; ICU: intensive care unit; IHCA: in-hospital cardiac arrest; LVEF: left ventricular ejection fraction; OR, odds ratio; ROSC: return of spontaneous circulation; RR: risk ratio; SGA: supraglottic airway device; VC defibrillation vector change defibrillation (pads switched into anterior–posterior position); VF: ventricular fibrillation.

## 4. Conclusions

As for all the medical sciences, also in the field of cardiac arrest, RCTs play a critical role in identifying effective interventions and improving outcomes for patients. The year 2022 was very prolific in terms of RCTs, with 11 articles published that covered almost all the links of the chain of survival, except for those focusing on high-quality CPR and on rehabilitation following hospital discharge. Interestingly, more than 80% of the RCTs encompassed advanced life support and post-cardiac arrest care. No studies focused on the treatment of IHCA, excluding one that had post-cardiac arrest TTM as a topic of interest. Surprisingly, all except two RCTs were neutral studies leading to equivalent results among all the new interventions tested in comparison to current practice. These RCTs, therefore, do not support any new intervention or treatment or ways to optimize the existing care of cardiac arrest patients, as expected when planned. Nevertheless, the results from these RCTs allow for a better understanding and improvement of the knowledge of the pathophysiology of cardiac arrest and provide solid data to generate new hypotheses to be tested in future studies. Interestingly, the EXACT trial [52] led to negative results, showing that early oxygen titration to obtain a target oxygen saturation of 90%–94% already in the pre-hospital setting decreased survival to hospital discharge and presented a higher rate of hypoxic episodes. The only RCT that provided positive results, despite the small sample size, was the DOSE VF trial [15] on new defibrillation strategies. This trial, designed on a solid pathophysiological basis, provided evidence that double sequential defibrillation and/or the change of the defibrillation vector may significantly improve the termination of VF and, ultimately, survival in refractory cardiac arrests. The results from this study have already led the International Liaison Committee on Resuscitation to draft a new consensus on science and treatment recommendations, suggesting new defibrillatory approaches in the instance of refractory VF (accessed on 6 March 2023: https://costr.ilcor.org/document/double-sequential-defibrillation-strategy-for-cardiac-arrest-with-refractory-shockable-rhythm-als-tf-sr).

## Data Availability

Not applicable.
